# The evolution of *Sex-linked barring* alleles in chickens involves both regulatory and coding changes in *CDKN2A*

**DOI:** 10.1371/journal.pgen.1006665

**Published:** 2017-04-07

**Authors:** Doreen Schwochow Thalmann, Henrik Ring, Elisabeth Sundström, Xiaofang Cao, Mårten Larsson, Susanne Kerje, Andrey Höglund, Jesper Fogelholm, Dominic Wright, Per Jemth, Finn Hallböök, Bertrand Bed’Hom, Ben Dorshorst, Michèle Tixier-Boichard, Leif Andersson

**Affiliations:** 1 Department of Animal Breeding and Genetics, Swedish University of Agricultural Sciences, Uppsala, Sweden; 2 GABI, INRA, AgroParisTech, Université Paris-Saclay, 78350 Jouy-en-Josas, France; 3 Department of Neuroscience, Uppsala University, Uppsala, Sweden; 4 Science for Life Laboratory, Department of Medical Biochemistry and Microbiology, Uppsala University, Uppsala, Sweden; 5 Linköping University, IFM Biologi, AVIAN Behavioural Genomics and Physiology group, Linköping, Sweden; 6 Department of Animal and Poultry Sciences, Virginia Tech, Blacksburg, Virginia, United States of America; 7 Department of Veterinary Integrative Biosciences, College of Veterinary Medicine and Biomedical Sciences, Texas A&M University, College Station, Texas, United States of America; National Institute of Health, UNITED STATES

## Abstract

Sex-linked barring is a fascinating plumage pattern in chickens recently shown to be associated with two non-coding and two missense mutations affecting the ARF transcript at the *CDKN2A* tumor suppressor locus. It however remained a mystery whether all four mutations are indeed causative and how they contribute to the barring phenotype. Here, we show that Sex-linked barring is genetically heterogeneous, and that the mutations form three functionally different variant alleles. The *B0* allele carries only the two non-coding changes and is associated with the most dilute barring pattern, whereas the *B1* and *B2* alleles carry both the two non-coding changes and one each of the two missense mutations causing the Sex-linked barring and Sex-linked dilution phenotypes, respectively. The data are consistent with evolution of alleles where the non-coding changes occurred first followed by the two missense mutations that resulted in a phenotype more appealing to humans. We show that one or both of the non-coding changes are *cis*-regulatory mutations causing a higher *CDKN2A* expression, whereas the missense mutations reduce the ability of ARF to interact with MDM2. Caspase assays for all genotypes revealed no apoptotic events and our results are consistent with a recent study indicating that the loss of melanocyte progenitors in Sex-linked barring in chicken is caused by premature differentiation and not apoptosis. Our results show that *CDKN2A* is a major locus driving the differentiation of avian melanocytes in a temporal and spatial manner.

## Author summary

Barring patterns on individual feathers are widespread phenomena in a number of wild bird species. Still, the genetic background and molecular mechanisms that give rise to barring remains poorly understood. Sex-linked barring is a striping pattern present on individual feathers in domestic chickens, which can be utilized as a model species to gain an understanding of the underlying ‘mode of action’ of biological pattern formation. Our findings suggest that regulatory mutations in the tumor suppressor gene *CDKN2A* first resulted in a primitive barring pattern and that two missense mutations in the same gene occurred later and independently, causing the more distinct barring pattern of extant chicken breeds. A plausible mechanism is that the altered expression of *CDKN2A* causes melanocyte progenitor cells to prematurely stop dividing and instead differentiate into pigment-producing cells. The temporary lack of melanocytes expresses itself as a white bar until the progenitor cells are replenished and pigment is produced to form a pigmented bar. It is remarkable that a good proportion of the world-wide production of animal protein is based on chicken that are carrying a functionally important missense mutation in the *CDKN2A* tumor suppressor gene.

## Introduction

**Fig 1:**
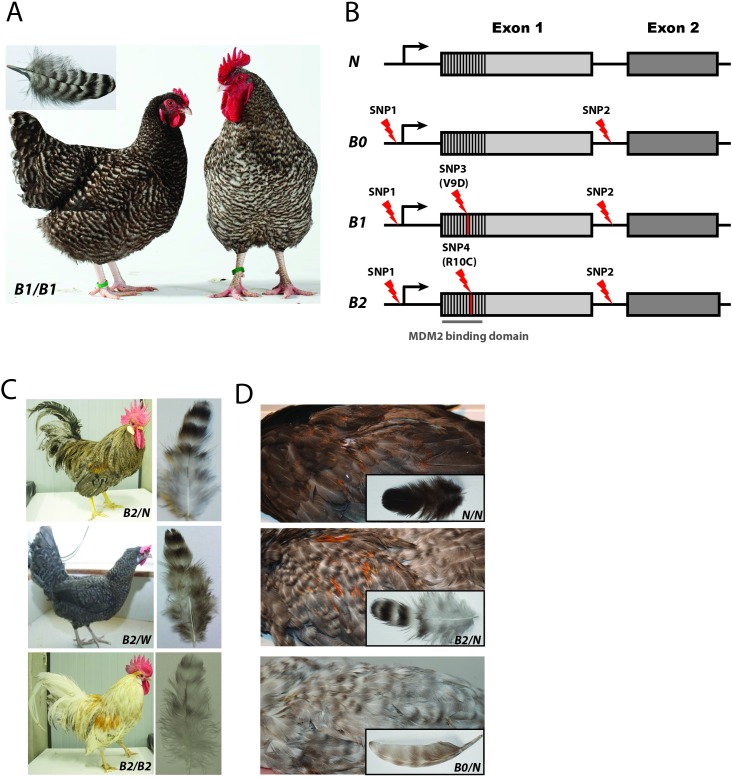
Alleles and phenotypes at the *Sex-linked barring* (*CDKN2A*) locus in chicken. (A) Female and male Coucou de Rennes chicken with separately depicted feather illustrating the iconic Sex-linked barring phenotype caused by the *B1* allele. (B) *Sex-linked barring* alleles and associated sequence variants. SNP1 and SNP2 are non-coding while SNP3 and SNP4 constitute non-synonymous changes in the region encoding the MDM2 binding domain. (C) Sex-linked dilution phenotype caused by the *B2* allele. Note how the homozygous male has an almost white appearance whereas the hemizygous female as well as the heterozygous male show a Sex-linked barring pattern. (D) Phenotype with individual feathers from *N/N*, *B2/N* and *B0/N* chicken. Photo credits: (A) Hervé Ronné, Ecomusée du pays de Rennes, (C) Susanne Kerje, (D) Dominic Wright and Doreen Schwochow-Thalmann.

Birds show an astonishing variety of plumage coloration and pattern, both across the body as well as on individual feathers. The phenotypic diversity in plumage color is due to the distribution of melanin (both eu- and pheomelanin), deposition of carotenoids (yellow and red colors), and structural colors caused by reflection, refraction and scattering of light in the feathers. The domestic chicken is a prime model species for exploring the underlying genetic mechanisms for variation in avian pigmentation due to the extensive plumage diversity that has accumulated since domestication. As it is more challenging to understand how color patterns are generated than to explain reductions or absence of pigmentation, barring is one of the most interesting feather patterns in chickens yet to be understood. Furthermore, barring in chicken resemble barring patterns that are common in wild birds. There are two different barring patterns in chicken, Autosomal and Sex-linked barring. Both barring patterns are characterized by alternating bars of two different colors on individual feathers. However, whereas chickens said to carry Autosomal barring, exhibit a black bar on a white or red background, feathers of Sex-linked barred chickens are characterized by a fully white bar on a red or black background (Fig 1A) [1]. Other characteristics of Sex-linked barring are the dilution of dermal pigment in the shanks and beak as well as a white spot on the head present at hatch (S1 Fig), which can be utilized for sex determination [2].

We have previously demonstrated that Sex-linked barring is controlled by dominant alleles at the *CDKN2A* locus, which encodes the alternate reading frame protein (ARF) [3]. We identified four SNPs located within a 12 kb region including *CDKN2A* exon 1 (Fig 1B). Two of the SNPs are present in non-coding regions, SNP1 in the promoter and SNP2 in intron 1. The other two SNPs are missense mutations; SNP3 causes a Valine to Aspartic acid (V9D) substitution while SNP4 causes an Arginine to Cysteine (R10C) substitution. These two neighboring residues are located in the binding site for the MDM2 (Mouse double minute 2 homolog) protein. The four mutations form three different alleles (Fig 1B), B*B0 (from now on referred to as B0), B*B1 (from now on referred to as B1) (Fig 1A), and B*B2 (from now on referred to as B2) (Fig 1C) and the wild-type allele at this locus is denoted B*N (N). As *CDKN2A* is located on the Z chromosome, male chickens can be either hetero- or homozygous for variant alleles, whereas females can only be hemizygous (e.g. *B1/W*). All three variant alleles carry the two non-coding mutations whereas V9D and R10C are associated with the *B1* and *B2* allele, respectively (Fig 1B). The *B1* allele determines the classical Sex-linked barring phenotype with sharp white and pigmented stripes also in homozygous birds as observed in Barred Plymouth Rock and Coucou de Rennes (Fig 1A). The *B2* allele corresponds to the *Sex-linked dilution* allele previously defined based on phenotype data [4, 5]. *B2/N* heterozygotes and *B2/W* hemizygotes show a clear barring phenotype whereas the *B2/B2* homozygotes show strong dilution of pigmentation to various degrees depending on the body region (Fig 1C and S2 Fig). The marked phenotypic difference between *B2/W* hemizygous females and *B2/B2* homozygous males illustrates the incomplete dosage compensation for sex-linked genes in birds. This allele also occurs in White Leghorn lines [3] and most likely contributes to pure white plumage in this breed. All Sex-linked barred chickens studied so far carry either the *B1* or *B2* alleles, whereas the phenotype associated with the *B0* allele remained unknown. In our previous study the *B0* allele was only found in White Leghorn chickens where the *Dominant white* allele (*I*), a strong dilutor of black pigment, prevents the observation of any patterning and is thus epistatic to *Sex-linked barring* [3]. However, the fact that the variant alleles at SNP1 and SNP2 were not found in any wild-type haplotype despite an extensive screening, suggested that they might be functionally important.

The finding that mutations in *CDKN2A* cause Sex-linked barring in chickens was unexpected as it is an important tumor suppressor gene that had not previously been associated with pigmentation phenotypes in any species. However, there is a strong link between *CDKN2A* and melanocyte biology as mutations inactivating the ARF protein are a major risk factor for familial forms of melanoma in humans [6–8]. In mammals, *CDKN2A* encodes two proteins (ARF and INK4A) via exon sharing [9]. Both proteins exhibit anti-proliferative properties, although mediated through different mechanisms by either activating p53 or interacting with the retinoblastoma protein [10]. ARF associates with a number of proteins promoting their posttranslational modification such as sumoylation and phosphorylation to activate or deactivate their function [11, 12]. Among those pathways, the one most frequently studied and best known, is the involvement of ARF in protecting the transcription factor p53 from degradation by binding to MDM2 [13, 14].

With only 60 amino acids (aa) the chicken ARF is substantially shorter than the human protein which comprises 132 aa [11] and there is only about a 35% overall sequence identity between chicken and mammalian ARF for the 60 shared residues [15]. Although studies do indicate that individual amino acid residues throughout the ARF protein can play an important role [16], the most N-terminal region (first 14 residues) seems to be functionally most important across species. In comparison to the rest of the protein, this region shows a relative high degree of sequence conservation between mammals and chicken and is implicated in nuclear localization, MDM2 binding, and the well-known role of ARF in inducing cell cycle arrest [11, 17]. Chicken ARF has been shown to interact with MDM2 and is able to protect the transcription factor p53 from degradation [15].

Melanocytes in both mammals and birds are derived from the neural crest and migrate during embryonic development to their biological destinations, mainly hair and feather follicles as well as the epidermal layer of the skin [18]. In the hair follicle, melanocyte progenitor cells that maintain their ability to divide are present in the hair bulge and give rise to fully functional melanocytes [19]. Similarly, in resting feathers quiescent melanocyte progenitor cells are present in a 3D ring at the base of the follicle and become activated in regenerating feathers [20]. The melanocyte progenitor cells start migrating up from the follicle base into the feather shaft and along the way become positive for a number of pigmentation markers as well as bigger in size and dendricity, indicating the differentiation of the pigment cell. Upon reaching the barbs, the progenitor cells become fully functional and pigment-producing melanocytes [20]. In contrast to avian melanocyte stem cells, mammalian melanocyte progenitor cells retained BrdU labeling almost 10 times longer, a finding that indicates that avian melanocyte stem cells cycle much more actively than the corresponding mammalian cells.

The aim of this study was (i) to determine if the *B0* allele, involving the non-coding SNP1 and SNP2 but none of the missense mutations, has a phenotypic effect and (ii) to explore the molecular mechanism causing Sex-linked barring. We show that *B0* has a more drastic effect on reducing pigmentation than *B1* and *B2*, and that the Sex-linked barring phenotype is caused by the combined effect of regulatory and coding mutations.

## Results

### The *B0* allele causes strong dilution of pigmentation

We crossed heterozygous *B0/B2* White Leghorn males, homozygous for the *Dominant white* allele *I/I*, with Red Junglefowl females (B*N/W, I*N/N). Doubly heterozygous males (either *B0/N* or *B2/N* and *I/N*) were then backcrossed to Red Junglefowl females. A total of 17 progeny carried the *Dominant white* allele (*I/N*) and were not informative. The 14 birds that were homozygous *N/N* or hemizygous *N/W* at the *B* locus were all non-barred as expected (Table 1). Three male progeny were heterozygous *B2/N* and exhibited Sex-linked barring. Eight males and four females were heterozygous or hemizygous for the *B0* allele and they showed a barring pattern that was markedly lighter than the more typical Sex-linked barring presented by *B2/N* birds (Fig 1D). No obvious difference in pigment intensity or bar spacing was observed between *B0/W* females and *B0/N* males. The phenotypic differences between *B0/*- (*B0/N* males or *B0/W* females) and *B2/-* (*B2/N* or *B2/W*) were already visible at hatch. Chicks carrying the *B2* allele were dark, almost black colored with a small light spot on the top of the head whereas *B0/-* chicks were much lighter with extended light spots both on the head as well as on the back (S1 Fig).

**Table 1:** Segregation of the Sex-linked barring phenotype in progenies from *B0/N* x *N/W* and *B2/N* x *N/W* matings. The progeny summarized here were all homozygous *wild-type* (*N/N*) at the *Dominant white* locus, which is epistatic to *Sex-linked barring*.

	Phenotype		
Genotype	Barred		Non-Barred
Male progeny	Light	Dark	
*N/N*	0	0	10
*B0/N*	8	0	0
*B2/N*	0	3	0
Female progeny			
*N/W*	0	0	4
*B0/W*	4	0	0
*Total*	12	3	14

### *CDKN2A* gene expression and allelic imbalance of expression in *B2/N* feathers

**Fig 2:**
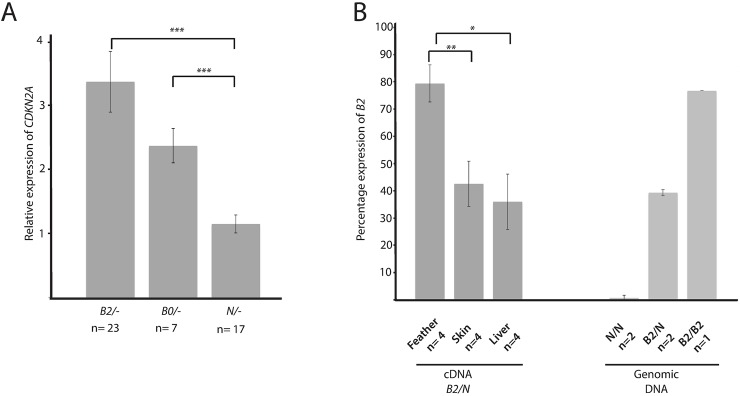
Differential expression of *CDKN2A* in feathers. (A) Relative expression of *CDKN2A* in Sex-linked barred chickens carrying the *B0* or *B2* allele and non-barred control feathers. Expression data was normalized using *EEF2* and *UB*. (B) Allele-specific expression of *CDKN2A* in *B2/N* feathers, skin and liver. Left panel: cDNA data using tissue samples from four *B2/N* chickens. Right panel: Genomic DNA from the different genotypes was used as control. The relative expression of the two alleles was determined by pyrosequencing. (Student’s t-test; * *P*<0.05, ** *P*<0.01, *** *P*<0.001).

The pedigree data, in combination with our previous genetic analysis [3], provided evidence that SNP1 and/or SNP2 are causing the strong phenotypic effects observed in *B0/N* and *B0/W* birds. This implies a regulatory change since both SNPs are non-coding (Fig 1B). We therefore analyzed the relative expression of *CDKN2A* in growing feathers in 7 *B0/-*, 23 *B2/-* and 17 non-barred (*N/-*) chickens. In both sets of barred feathers, *CDKN2A* was on average 2.5 times (*B0/-*) or 3.3 (*B2/-*) times higher expressed as compared to the control samples (Fig 2A; Student’s t-test, *P* = 3.6x10^-4^ and *P* = 3.2x10^-5^, respectively). There was no statistically significant difference in *CDKN2A* expression between the two barring genotypes (Student’s t-test, *P* = 0.4).

To verify that the observed differential expression is caused by altered *cis*-regulation, we evaluated the relative expression of the *B2* and *wild-type* alleles within heterozygous *B2/N* chickens. As the *B2* allele harbors both the two non-coding mutations as well as one of the missense mutations in the first exon of *CDKN2A*, this coding SNP can be used to distinguish the relative expression of the two alleles. For this purpose we reverse transcribed RNA obtained from different tissues (feather, skin and liver) of *B2/N* chickens and sequenced them using pyro-sequencing. The pyrograms obtained from four heterozygous chickens consistently showed a higher peak for the *B2* allele in feathers (x = 79.4±6.8%; Fig 2B) and was statistically different from the one observed in liver (x = 42.6±8.3%) and skin (x = 36±10.2%; Student’s t-test, *P* = 0.03 and *P* = 0.003, respectively). In liver and skin the two alleles did not show a significant allelic imbalance since the *B2/N* ratio was very similar to the one found in genomic control DNA for *B2/N* heterozygotes where the copy number should be 50:50. We did not successfully amplify any *CDKN2A* transcripts from muscle tissue.

Previous work has shown that ARF is involved in protecting the transcription factor p53 from ubiquitination and degradation by binding to MDM2 [13, 14]. An altered expression of ARF could therefore affect expression of p53 downstream targets. We therefore evaluated the expression of four genes involved in cell cycle regulation and apoptosis: Bcl2 associated X protein (*BAX*), Cyclin-dependent kinase inhibitor 1 A (alias p21) (*CDKN1A*), damage-regulated autophagy modulator 1 (*DRAM1*) as well as Pleckstrin homology-like domain, family A, member 3 (*PHDLA3*). We were able to detect expression of all genes under the described conditions except for *BAX*, but only *PHLDA3* showed a marginally significant higher expression in *B0/-* feathers (Student’s t-test, *P* = 0.03; S3 Fig). Since ARF is interacting with a number of proteins other than p53 that could directly or indirectly be involved in cell cycle regulation, we also examined the relative RNA expression levels of four members of the 14-3-3 gene family involved in cell cycle arrest regulation using the same sample set as used for p53 downstream targets: Tyrosine 3-monooxygenase/tryptophan 5-monooxygenase activation protein beta (*YWHAB*), Tyrosine 3-monooxygenase/tryptophan 5-monooxygenase activation protein epsilon (*YWHAE*), Tyrosine 3-monooxygenase/tryptophan 5-monooxygenase activation protein zeta (*YWHAZ*) and Stratifin (*SFN*). Each target gene was normalized with two housekeeping genes stably expressed in the respective tissue. *YWHAB* showed lower relative expression in mutant feathers compared to non-barred feathers (Student’s t-test, *P* = 0.03; S3 Fig) but no other member of this gene family showed a statistically significant differential expression. This suggestive downstream effect on *YWHAB* expression needs to be further corroborated in new samples where the specific stage of feather development is better defined than was available in the current study.

To summarize, *CDKN2A* shows higher expression both in *B0/-* and *B2/-* barred feathers than in *wild-type* feathers, which might affect downstream targets of p53 like *PHLDA3* or cell cycle regulation genes such as *YWHAB*. A possible explanation for our failure to detect stronger significant changes in *CDKN2A* downstream targets could very well be that only a small proportion of cells in the feather follicle show up-regulated expression of *CDKN2A* (see below). Furthermore, we observed allelic imbalance in favor of the mutant allele in *B2/N* birds suggesting that one or both non-coding changes act *cis*-regulatory. As we did not observe statistically significant differences in *CDKN2A* expression between the *B0/-* and *B2/-* genotypes, we conclude that the coding changes do not have any major effect on gene expression.

### Immunohistochemistry (IHC) and *in situ* hybridization (ISH) in feather follicles

During active growth of a feather, a 3-D ring of melanocyte progenitor cells residing on the bottom of the feather follicle becomes activated and migrates upwards through the bulge region, the ramogenic zone and eventually reaches the barbs where they become fully differentiated and start producing pigment. ARF might play a critical role in this developmental process as it affects the stability of the transcription factor p53 [13, 14], which activates a number of downstream targets leading to either cell cycle arrest or apoptosis. In order to distinguish these two processes we used IHC to assess the presence of cleaved Caspase-3 proteins, which are indicative of apoptosis. In all growing feather follicles irrespective of genotype (*B2/N* n = 4, *B0/W* n = 4, and *N/N* n = 4), many Caspase-3 positive cells were observed in the pulp region of the feathers all the way across from the papilla ectoderm to the barb region (S4 Fig). No positive cells were observed in the barb region for any genotype. We therefore conclude that melanocytes were not in a pre-apoptotic state in any feather part.

**Fig 3:**
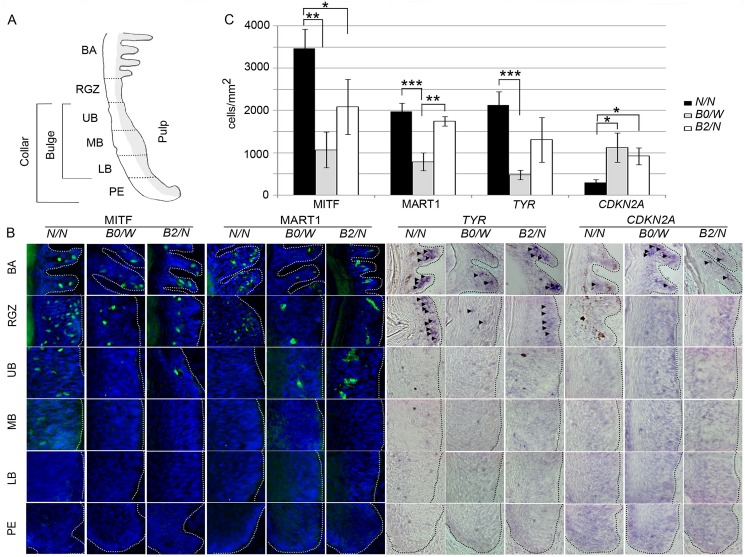
Characterization of the expression of MITF, MART1, *TYR* and *CDKN2A* in melanocyte progenitors and differentiated pigment cells in feathers from different genotypes at the *Sex-linked barring* locus. (A) Anatomy of growing feather, papilla ectoderm (PE), lower bulge (LB), middle bulge (MB), upper bulge (UB), ramogenic zone (RGZ), and barb (BA). (B) Distribution of cells from the melanocyte lineage across different parts of the feather in different genotypes detected by immunohistochemistry (MITF and MART) and *in-situ* hybridization (*TYR* and *CDKN2A*). (C) Average number of MITF+, MART1+, *TYR+* and *CDKN2A+* positive cells in the barbs of chickens with different genotypes. Significant differences are indicated by stars (One-way ANOVA, Tukey’s multi-comparison post-hoc test; * *P*<0.05, ** *P*<0.01, *** *P*<0.001).

Using the melanocyte marker Microphthalmia-associated transcription factor (MITF), IHC revealed that MITF+ cells first appeared in the lower bulge (LB) region in wild-type (*N/N*) feathers (although they were more commonly observed in the middle bulge (MB) region), in the upper bulge (UB) region in *B2/N* chicken, whereas in feathers from all four *B0/W* chicken MITF+ cells were not observed until the ramogenic zone (RGZ) just below the barbs (Fig 3, S1 Table). The number of MITF+ cells increased towards the barbs (BA) in all genotypes. Statistical analysis revealed significant differences in the numbers of MITF+ cells in the barbs among the three genotypes (Fig 3C; One-way ANOVA, Tukey’s multi-comparison post-hoc test, *P*<0.01 and *P*<0.05 for *N/N* vs. *B0/W* and *N/N* vs. *B2/N*, respectively). Whereas the average number of cells expressing MITF+ protein/mm^2^ reached almost 3470±454 in *N/N* chickens, the corresponding numbers for *B0/W* and *B2/N* birds were only 1070±419 and 2090±648, respectively (S1 Table). The trend for Melanoma antigen recognized by T-cells 1 (MART1; Fig 3, S1 Table) was quite similar to MITF, but there was a tendency that MART1+ cells appeared further down in the feather follicle in *B0/N* or *B2/W* chicken compared to *wild-type*. Starting from UB to the barbs the number of MART1+ cells increased steadily for all genotypes, reaching on average 1970±207 cells/mm^2^ in *N/N*, 1740±109 in *B2/N* and less than 790±209 in *B0/W* chicken, a difference which again reach statistical significance (Fig 3C; One-way ANOVA, Tukey’s multi-comparison post-hoc test, *P*<0.001 and *P*<0.01 for *N/N* vs. *B0/W* and *B0/W* vs. *B2/N*, respectively).

Fully pigmented wild-type cells were first observed in the RGZ, which corresponded to the results of the ISH analysis of Tyrosinase gene (*TYR*), encoding the rate-limiting enzyme in pigment production (Fig 3, S1 Table). In *B0/W* chickens, few *TYR*+ cells were present in the UB region of the feather and the number of these cells just slightly increased towards the barbs and reached on average 481±113 cells/mm^2^ whereas in wild-type chickens more than 2130±323 *TYR*+ cells/mm^2^ were counted. The number of *TYR*+ cells in the barbs of *B2/N* birds was almost three-fold higher than in *B0/W* birds but still remained at about 50% of those seen in wild-type chickens (Fig 3C; One-way ANOVA, Tukey’s multi-comparison post-hoc test, *P*<0.001 for *N/N* vs. *B0/W*). Whereas we could observe an increase of cells expressing any of the melanocyte-specific markers from UB to the barbs, the opposite trend was true in *CDKN2A*+ cells (Fig 3, S1 Table). In the RGZ, *CDKN2A*+ cells were only present in the mutant genotypes and in the barb region the number of *CDKN2A*+ positive cells in *B0/W* and *B2/N* birds were significantly higher than in wild-type birds (Fig 3C; One-way ANOVA, Tukey’s multi-comparison post-hoc test, *P*<0.05). The signal intensity for the *CDKN2A in situ* probe was quite weak and we measured the signal intensity per cell to test the possibility that mutant birds showed higher expression and therefore a larger number of *CDKN2A*+ cells were called in the mutant genotypes. However, this analysis did not reveal any significant difference in signal intensity among the three *CDKN2A* genotypes (S2 Table).

In summary, we observed a reduction in total pigment cell numbers in the barbs of Sex-linked barred chickens compared to the wild-type. The reduction was already visible in lower parts of the feather shaft and was most prominent in *B0/-* feathers. The opposite trend is true for *CDKN2A*, which is expressed earlier in the melanocyte migration process in feathers from *B0/-* and *B2/-* birds. Furthermore, compared to pigment cells in the same region of wild-type chickens, *B0/W* and *B2/N* pigment cells appeared more dendritic-like and more closely resembled mature melanocytes already below the barbs.

### The coding mutations impair ARF–MDM2 interaction

**Fig 4:**
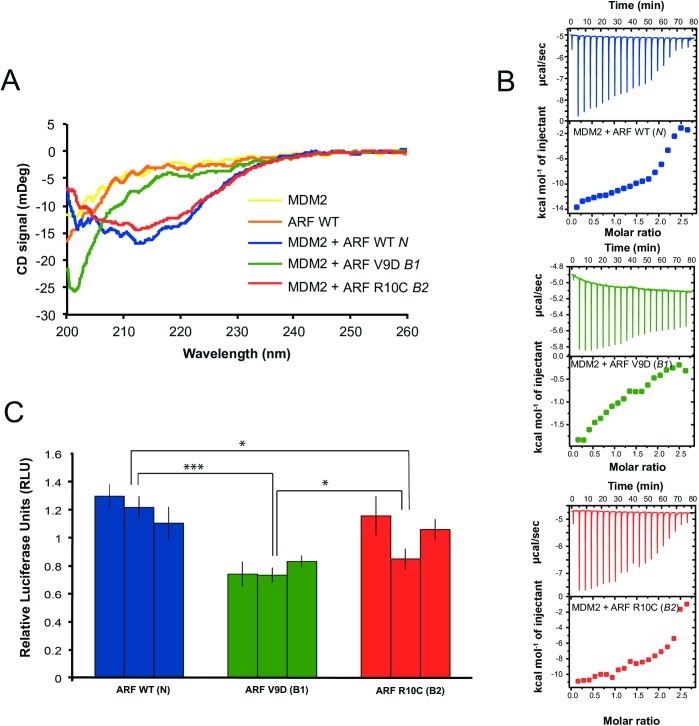
Functional characterization of the V9D and R10C substitutions in chicken ARF. (A) Far-UV circular dichroism (CD) is measured in mDeg and monitors protein secondary structure. Spectra of MDM2_204-298_, ARF_1-14_^WT^, and complexes of ARF_1-14_^WT^/MDM2_204-298_, ARF_1-14_^V9D^/MDM2_204-298_, and ARF_1-14_^R10C^/MDM2_204-298_. MDM2_204-298_ was used at 10 μM and all peptides at 64 μM final concentrations. Spectra of all peptides in free form and at 25 μM concentration in complex with MDM2_204-298_ are shown in S5 Fig. (B) Isothermal titration calorimetry (ITC) experiments in which the heat (enthalpy) associated with binding is recorded as μcal/sec for each titration point (top panel) and integrated and normalized against the concentrations of ARF peptide and MDM2_204-298_ to obtain a binding isotherm (expressed as kcal mol^-1^ versus molar ratio; bottom panel). In the experiment, ARF peptides (WT, V9D, and R10C, respectively) were titrated into 100 μM MDM2_204-298_ with the peptide concentration increasing by approximately 10 μM in each titration point. Peaks (top) and integrated energies (bottom) corrected for heats of dilution are shown. Raw data are shown in S6 Fig. (C) Assessment of the effect of the two coding mutations on the interaction between ARF and MDM2 based on a luciferase assay. Reduced luciferase activity implies weaker interaction between ARF and MDM2, monitored as a decreased ability of ARF to protect the transcription factor p53 from degradation (Student’s t-test; *P<0.05, ***P<0.001).

The striking phenotypic differences associated with the *B0*, *B1*, and *B2* alleles imply that the two missense mutations must affect ARF function since all three alleles share the two non-coding changes associated with Sex-linked barring (Fig 1). To learn more about the specific effects of the coding ARF mutations we performed biophysical studies on peptides corresponding to the N-terminus of wild-type and mutant chicken ARF (ARF_1-14_^WT^, ARF_1-14_^V9D^, ARF_1-14_^R10C^) and purified chicken MDM2_204-298_. Previously, using a combination of ultracentrifugation, far-UV circular dichroism (CD) and NMR experiments, it was shown that the mammalian ARF N-terminus and MDM2_210-304_ (human MDM2 NCBI accession number XP_005268929; corresponding to chicken MDM2_204-298_) are both intrinsically disordered in their free states and interact by forming an oligomeric β-structure [21, 22]. While the structure of the ARF/MDM2 complex was not determined, its formation could easily be detected by far-UV CD, which is sensitive to optically active chiral molecules and thus can monitor protein secondary structure. We therefore performed CD experiments with different combinations of chicken MDM2_204-298_ and the ARF_1-14_ peptides. MDM2_204-298_ in buffer yielded a CD spectrum consistent with an intrinsically disordered protein, and so did the ARF_1-14_ peptides (Figs 4A and S5). However, when MDM2_204-298_ was mixed with wild-type ARF_1-14_^WT^ peptide the spectrum adopted a shape strikingly similar to that of the mammalian ARF/MDM2 complex, and thus consistent with formation of a β-structure (Figs 4A and S5A). Moreover, the ARF_1-14_^R10C^ peptide produced a similar spectrum as ARF_1-14_^WT^ upon mixing with MDM2_204-298_, but it required a higher peptide concentration to fully recapitulate the ARF_1-14_^WT^ spectrum (Figs 4A and S5C). On the other hand, the spectrum with the ARF_1-14_^V9D^ peptide showed no evidence of secondary structure formation suggesting it did not bind to MDM2_204-298_ at the concentrations used in the CD experiment (Figs 4A and S5B).

To corroborate the results from the CD experiments we pursued isothermal titration calorimetry (ITC) experiments with MDM2_204-298_ and ARF_1-14_ peptides. ITC measures the change in enthalpy (heat) for any reaction and is therefore perfectly suited for studying protein-ligand interactions. For each injection of ligand into the protein solution the change in heat is recorded and subsequently analyzed with a suitable model, which in the simplest case is a 1:1 binding. The data clearly show that two of the interactions, ARF_1-14_^WT^/MDM2_204-298_ and ARF_1-14_^R10C^/MDM2_204-298_, respectively, are qualitatively similar (Figs 4B, S6A and S6C). Moreover, the non-sigmoidal shape of the integrated peaks for these two interactions suggest that they do not follow a simple 1:1 binding mechanism and were therefore not quantitatively analyzed. The complex binding isotherm is consistent with the β-structure model proposed for mammalian ARF/MDM2 [21, 22]. In contrast to CD, the ITC experiment of ARF_1-14_^V9D^/MDM2_204-298_ suggested an interaction, but the titration profile was distinct from those of ARF_1-14_^WT^ and ARF_1-14_^R10C^ and reflected a binding event of lower affinity (Figs 4B and S6B). The detection of an interaction between ARF_1-14_^V9D^/MDM2_204-298_ with ITC but not CD can be attributed to the greater total concentrations of MDM2_204-298_ and ARF_1-14_ peptide in the ITC experiment as well as higher sensitivity of ITC in comparison with CD. Thus, the data from ITC and CD agree well.

As our data show that the mutations in chicken ARF affect its direct interaction with MDM2, we decided to explore if this effect is translated further downstream on the ability of ARF to protect p53 from degradation. We utilized a previously described luciferase assay involving a p53 binding promoter element and transient transfection of U2OS cells (which do not express INK4a and ARF proteins) [16]. If the cells expressed the chicken ARF version containing the mutation V9D as harbored in the *B1* allele, the ability of p53 to activate the luciferase promoter was decreased to on average 58% compared to the *wild-type* (Fig 4C, *P* = 0.001). A similar picture was observed for R10C (as in the *B2* allele), although with a slightly less obvious trend (on average 77%, *P* = 0.03). The activity of V9D- and R10C- was significantly different (*P* = 0.04).

In conclusion, our biophysical data showed that chicken ARF_1-14_^WT^ and MDM2_204-298_ interact, likely by forming a β-sheet structure similar to that of mammalian ARF/MDM2 [21, 22]. Furthermore, both the luciferase as well as the ITC/CD experiments showed a similar trend consistent with a more severe effect of the V9D mutation, which appears to be more disruptive resulting in much lower affinity for MDM2, whereas the R10C mutant of ARF shows a similar behavior as ARF_1-14_^WT^ but with slightly lower apparent affinity. This trend was corroborated with the results by the luciferase assay.

## Discussion

Our previous study provided conclusive evidence that Sex-linked barring is controlled by mutations in *CDKN2A* and that two different non-synonymous substitutions (V9D and R10C) are associated with two different alleles (*B1* and *B2*, respectively) at this locus [3]. However, this previous study left unanswered the enigma of why both missense mutations were associated with two non-coding changes that were not detected in any sequenced *wild-type* haplotype. Thus, the two missense mutations must have occurred on the very rare haplotype containing the two non-coding variants. We have now resolved this enigma by showing that the *B0* allele, carrying only the two non-coding changes (Fig 1B), causes a more extreme reduction of pigmentation than the *B1* and *B2* alleles (Fig 1D). This implies that at least one or both of the non-coding changes constitute *cis*-acting regulatory mutation(s); if only one is causal, the other has hitchhiked with the causal mutation on this haplotype. This hypothesis was supported by the observed up-regulated expression of *CDKN2A* in Sex-linked barred feathers as well as the observed allelic imbalance with higher expression of the *B2* allele in *B2/N* heterozygotes in growing feathers (Fig 2). We also show that this up-regulated expression is highly tissue-specific since it was observed in feather follicles but not in skin and liver.

We propose that the *Sex-linked barring* locus is composed of four alleles with distinct phenotypic effects: *N*, *wild-type*; *B0*, *Sex-linked extreme dilution*; *B1*, *Sex-linked barring*; and *B2*, *Sex-linked dilution*. The *B0* allele was first defined in our previous study based on sequence data [3] and we now document that it in fact has the strongest effect on pigmentation (Fig 1D). Therefore we propose the name *Sex-linked extreme dilution*. This allele has so far only been found in White Leghorn chickens and we have not yet observed the phenotype of *B0/B0* homozygotes in the absence of the epistatic *Dominant white* allele, but we assume that these birds have very little pigmentation.

**Fig 5:**
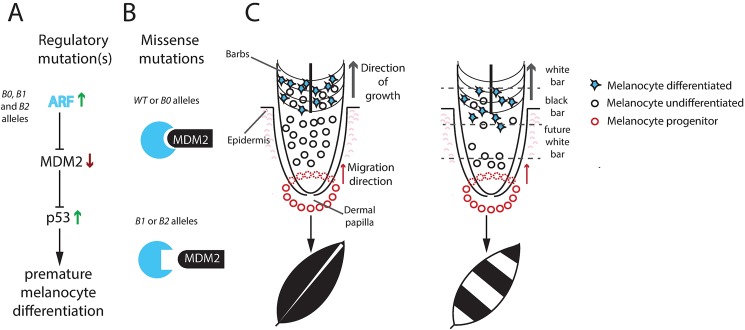
Proposed mechanism for development of the Sex-linked barring phenotype. (A) The non-coding mutation(s) present in the *B0*, *B1* and *B2* allele cause a tissue specific up-regulation of *CDKN2A* encoding the ARF protein. ARF inhibits MDM2-mediated degradation of p53. p53 will activate downstream targets possibly initiating premature melanocyte differentiation and thereby loss of mature pigment cells. (B) The missense mutations present in the *B1* and *B2* alleles impair the interaction between ARF and MDM2, which counteract the consequences of up-regulated ARF expression. (C) In solid colored feathers, melanocyte progenitor cells migrate up from the feather base and start expressing *CDKN2A* in the barb region leading to differentiation of melanocytes and pigment production without exhausting the pool of undifferentiated melanocytes. In sex-linked barred feathers, up-regulated ARF expression may lead to premature differentiation of pigment cells and a lack of undifferentiated melanocytes that can replenish the ones producing pigment. As the feather keeps on growing, no more melanocytes are available to produce pigment resulting in the white bar. A plausible explanation for the cyclic appearance of white and black bars is that new recruitment of melanocyte progenitor cells takes place after the undifferentiated melanocytes have been depleted.

Available phenotypic data indicate a ranking of the three variant alleles regarding pigment reduction, as follows: *Sex-linked extreme dilution* > *Sex-linked dilution* > *Sex-linked barring*. Our functional data are fully consistent with the proposed ranking. Firstly, expression analysis shows that one or both of the non-coding changes cause an up-regulation of *CDKN2A* expression in feather follicles during feather growth (Fig 5A). A higher expression of ARF, encoded by *CDKN2A*, is expected to lead to a reduction of pigment cells due to apoptosis, cell cycle arrest or premature differentiation of melanocytes. The *Sex-linked extreme dilution* (*B0*) allele carries only these non-coding changes and is associated with a drastic reduction in pigmentation. In contrast, our three functional assays (CD, ITC, and luciferase reporter assay) all indicate that the two missense mutations (V9D and R10C) result in hypomorphic ARF alleles. Thus, these mutations are expected to counteract the effect of up-regulated ARF expression, most likely by impairing the ARF-MDM2 interaction and thereby lead to a less severe reduction in pigmentation (Fig 5B). Furthermore, the three functional assays all indicate that the V9D substitution (*B1*) is expected to limit the effect of the non-coding mutations (*B0*) on pigment dilution to a larger extent than does R10C (*B2*), which is consistent with the observation that *Sex-linked dilution* (*B2*) shows a stronger reduction in pigmentation than does *Sex-linked barring* (*B1*), at least in the homozygous condition (Fig 1A and 1C).

Our results imply an evolutionary scenario where the non-coding change(s) occurred first and resulted in *Sex-linked extreme dilution* (*B0*). This was followed by the independent occurrence of the two missense mutations resulting in the appearance of *Sex-linked barring* (*B1*) and *Sex-linked dilution* (*B2*) alleles. The latter two alleles are much more widespread among chicken breeds [3] and it is likely that the missense mutations have been under strong positive selection simply because they generated phenotypes more appealing to humans as illustrated by the iconic Barred Plymouth Rock chicken or the French breed Coucou de Rennes (Fig 1A). The *Sex-linked barring* locus is another striking example of the ‘evolution of alleles’ that has occurred in domestic animals by the accumulation of multiple causal mutations affecting the same gene [23]. Other examples include dominant white color in pigs [24], dominant white/smoky plumage color in chicken [25], rose-comb in chicken [26] and white spotting in dogs [27]. It is very likely that allelic variants differing by multiple causal changes are common in natural populations. An excellent candidate for this scenario is the *ALX1* haplotype associated with blunt beaks in Darwin’s finches [28]. This haplotype is associated with derived changes at two highly conserved amino acid residues as well as changes at highly conserved non-coding sites. The beauty with studying the ‘evolution of alleles’ in domestic animals, as illustrated in our study, is that the phenotypic consequences of the intermediate steps in such a process can be revealed due to the relatively short evolutionary history of domestic animals.

A previous study on Sex-linked barring in chickens suggested that the alternate barring pattern might be caused by premature apoptosis as a result of the gain-of-function mutations in *CDNK2A* [3]. This was based on the finding that no pigment cells are present in the feather during white band formations [29, 30] as well as the observation that melanocytes from Barred Plymouth Rock chickens in cell culture die five times earlier than wild-type melanocytes [31]. More recently, Lin et al. [20] proposed, based on negative TUNEL staining in growing feathers from Sex-linked barred chickens, that the lack of melanocytes in the white bar is not attributed to apoptosis but to premature differentiation of melanocytes. Our result is fully consistent with this hypothesis since our Caspase-3 assay did not reveal any apoptotic melanocytes or precursors in any feather region of any mutant phenotype tested. The only cells that were expressing Caspase-3 proteins, were located in the middle of the feather shaft, the pulp region, and represent a population of keratinocytes which will disappear after the feather has finished growing, leaving behind a hollow skin structure [32].

When we examined the presence of melanocyte progenitor and melanocyte cells in the feather follicles of both mutant and wild-type chickens, it became clear that fewer cells expressed MART1 and Tyrosinase (*TYR*) in feathers from mutant birds (*B2/N* and *B0/W*). The cells of mutant birds also appeared more dendritic and more closely resembled mature melanocytes at a much earlier stage of development. This suggests that feather melanocytes from mutant birds (*B0*, *B1*, *B2*) reach a more mature state at an earlier point in migration as compared to the wild-type. Although we were not able to directly follow individual cell maturation through their migration in the feather follicle, we did observe expression of *CDKN2A* mRNA in mutant birds already in the ramogenic zone of the feather follicle. This result is consistent with the recently proposed model that loss of melanocyte progenitors in Sex-linked barred chicken is caused by premature differentiation and not apoptosis [20]. This mechanism implies that the melanocyte progenitor pool in Sex-linked barred birds becomes smaller while the cells are migrating up the feather shaft, leading to fewer pigment-producing cells towards the end of the formation of the pigmented bar. Eventually, the progenitor pool becomes exhausted causing a complete lack of pigment-producing cells and the formation of the white bar (Fig 5C). The melanocyte progenitor cells are later replenished by a new pool of undifferentiated cells, migrating up from the feather base and leading to pigment production as well as the formation of the next pigmented bar. As the feather continues to grow, the cyclic behavior of the melanocyte progenitor cells leads to a periodic striping pattern.

Despite mutant birds had a reduced number of cells of the melanocyte lineage in the upper feather shaft region, they had more *CDKN2A*+ cells as compared to the wild-type. This result suggested that the increased *CDKN2A* expression detected by RT-qPCR (Fig 2A) could be due to an increased number of cells expressing *CDKN2A* rather than higher expression per cell. In fact, measurement of the *in situ* hybridization signal intensity for *CDKN2A* revealed no significant difference in expression level per cell among genotypes. How is this result compatible with the highly significant allelic imbalance test showing that the mutant allele is expressed at a higher level than the wild-type allele in feather follicles (Fig 2B)? One possible explanation is that the wild-type allele is down-regulated in the presence of a highly expressed mutant *CDKN2A* allele. However, this appears unlikely since that would indicate that there should be no phenotypic effects in birds carrying the *B0* allele, which express an ARF wild-type form at the protein level. The striking phenotypic effects associated with this allele as regards the increased number of *CDKN2A*-positive cells, fewer pigment cells and reduced pigmentation must be mediated through altered gene regulation. Therefore, a more plausible explanation is that the *CDKN2A*-positive cells detected in the upper feather shaft region are a mixed population. In mutant birds a fraction of these solely or predominantly expresses the mutant *CDKN2A* allele possibly explaining the observed allelic imbalance. This can be addressed in future studies by double labeling experiments or single cell transcriptomics.

Although *CDKN2A* is expressed in most tissues and has an important role in cell cycle regulation, there is a fascinating tissue-specificity of the phenotypic effects of both the human loss-of-function mutations and the chicken mutations, suggesting that melanocytes are particularly sensitive for changes in ARF function. *CDKN2A* loss-of-function mutations are associated with a high risk of melanoma in humans but not for other tumor forms [6–8]. Similarly, the chicken *CDKN2A* alleles have a striking effect on pigmentation, but no or only minor pleiotropic effects on other tissues. Our expression data indicate that the regulatory mutations have a very tissue-specific effect in feather follicles whereas the missense mutations will affect ARF function in all cells that express this protein. It is remarkable that a good proportion of the world-wide production of animal protein is based on chickens carrying *CDKN2A* alleles involving the V9D or R10C missense mutations, resulting in hypomorphic ARF proteins. These include white-egg layers, i.e. White Leghorn, and many commercial broiler lines used for meat production, most of them having a white plumage. We assume that the overall protein interaction ability of the mutated chicken ARF is still sufficient to maintain its function in other tissues. Alternatively, the missense mutations could only affect a pathway specific to the feather follicle or the ARF-dependent activation of p53-dependent cell cycle arrest may be mediated by other structural parts of ARF or even by a completely different protein in other cell types. However, since domestication is an evolutionary process, it is possible that these mutant *CDKN2A* alleles initially had slightly deleterious effects but that genetic modifiers counteracting the effects of the ARF mutations have accumulated over time.

## Materials and methods

### Animals

Tissue samples and phenotype data were obtained from either an F_8_ generation of a pedigree originally used to map *Sex-linked barring* [3] as well as a separate backcross which was set up involving four White Leghorn L13 males heterozygous *B0/B2* (for breed pedigree details see [33]). These birds were first crossed with four Red Junglefowl females hemizygous *N/W*. Four F_1_ males were heterozygous *B0/N* and three were heterozygous *B2/N*. All F_1_ males were also heterozygous for the Dominant White mutation, *I/N*, (fixed in the WL L13 population, and absent from the Red Junglefowl population). Each F_1_ male was then back-crossed to a Red Junglefowl female, with a total of 48 back-cross offspring. These birds were genotyped for *B* and *I* and phenotyped at hatch as well as soon as the adult plumage was apparent (at 49 days). Approval from the Ethical Committee for animal experiments in Uppsala, Sweden was obtained for all experiments involving the F_8_–generation chickens (2011-11-25, C307/11). The experiment involving the F_2_ barring intercross was approved by the Regional Committee for Ethical Approval of Animal Experiments (Swedish Board of Agriculture DNR# 122–10). All animals were handled by trained personnel and reared according to the guidelines of the Swedish Board of Agriculture for chickens.

### Sample collection

For RNA extraction, growing feathers of different sizes were either collected from different parts of the body where they occurred naturally and which showed a Sex-linked barring phenotype or from regions, which were plucked seven to ten days before (i.e. in the neck). Skin and muscle samples were obtained from the back and the breast respectively and, just like the liver samples, collected right after euthanasia of the animal using 1 ml of 100 mg/ml Thiopental Inresa/kg body weight (Inresa Arzeneimittel GmbH) or cervical vertebra dislocation. The feather shafts and tissue samples were shock-frozen on dry ice or liquid nitrogen immediately after collection and stored at -80°C until further processing.

Skin tissue samples including feathers used for *in situ* hybridization (ISH) or immunohistochemistry (IHC) were first fixed in 4% paraformaldehyde in phosphate-buffered saline for 1 h (ISH) or 15 min (IHC) at 4°C. The tissue samples were then incubated over night (ISH) or for 3 h (IHC) in 30% phosphate-buffered sucrose at 4°C, embedded in Neg-50 frozen section medium (Thermo Fisher Scientific), frozen and sectioned in a cryostat. Ten μm thick sections were collected on glass slides (Super Frost Plus; Menzel-Gläser, Menzel GmbH & Co KG).

### RNA extraction and cDNA synthesis

One or two frozen feather shafts or small pieces of liver or skin tissue were removed from the storage tube and transferred to an RNase free tube containing Zirconia beads (Biospec Products) and 1 ml of TRIzol (LifeTechnologies). Feather, liver and muscle samples were homogenized immediately using a Mini-Beadbeater (Biospec Products) at highest speed for 20 s up to 1 min depending on the softness of the tissue. For skin, a Precellys24 Tissue homogenizer (Bertin Technologies) was used at 6800 rpm/min for 30 s intervals until the samples were properly homogenized. The homogenate was centrifuged 5 min at 2,000 x g and 4°C and the supernatant transferred to a new RNase free 1.5 ml Eppendorf tube. Skin samples were subjected to an additional isolation step. Following homogenization, the samples were centrifuged at 12,000 x g for 10 min at 4°C. The cleared supernatant was transferred to a new tube. The TRIzol volume was adjusted to 1 ml, 0.2 ml of Chloroform (Sigma-Aldrich) were added and the sample tube properly mixed using a Vortexer (Scientific Industries, Inc). The mixture was incubated at room temperature for 2 min to allow for phase separation. To obtain the aqueous phase containing the RNA, the samples were centrifuged at 12,000 x g for 15 min (4°C) and the upper phase was transferred to a new tube. An equal volume of 70% ethanol was added to the upper-phase solution and 0.7 ml of the mixture applied to a PureLink RNA Spin Cartridge (PureLinkRNA Mini Kit, LifeTechnologies) and centrifuged at 12,000 x g for 15 s. The purification procedure followed the manufacturer’s recommendation (PureLinkRNA Mini Kit, LifeTechnologies), including DNase treatment of each sample. The muscle tissue samples were homogenized using Zirconia beads as described above in 300 μl lysis buffer containing 1% β-Mercaptoethanol (Sigma-Aldrich). The samples were either kept at -80°C until further use or processed immediately using the RNeasy Fibrous Tissue Mini Kit (Qiagen) according to the manufacturer’s protocol with the following modification: samples were incubated with 10 μl proteinase K solution for 7 min at 55°C, then another 10 μl proteinase K aliquot was added and incubated for additional 7 min. The eluted RNA was evaluated for its quantity and quality using a NanoDrop- 1000 Spectrophotometer (Thermo Scientific) and stored at -80°C until further use. RNA samples were treated again with DNase using the DNAfree Kit (Life Technologies) according to the manufacturer’s suggestions with the following modifications: 1 μl of DNase was used before the 30 min incubation and after 15 min at 37°C an additional 1 μl of DNase was added to each tube.

DNA-free RNA samples were reversed transcribed using the RT-PCR protocol and reagents for the Maxima H Minus First Strand cDNA Synthesis Kit (Thermo Scientific). In step one, 1 μl of 1:1 Oligo (dT)18 primer/random hexamer mixture was used. The samples were incubated as follows: 10 min at 25°C, 30 min at 50°C and finally 5 min at 85°C to inactivate the enzyme. The obtained cDNA was used right away for qPCR or pyro-sequencing or temporary stored at -20°C.

### DNA extraction

DNA samples for genotyping were either obtained from blood routinely collected as a part of monitoring and documenting information on the chicken crosses or from muscle samples after euthanasia of the animal. DNA from blood was extracted using standard salting-out methods [34] whereas DNA from muscle tissue was obtained using the DNeasy Blood and Tissue Kit (Qiagen).

### Expression analysis

Primers were designed using Primer3Plus software [35] and the PCR products were checked for possible formations of secondary structures using the online tool mFold (http://mfold.rna.albany.edu/?q=mfold). Before usage in the actual assay, the primers were tested for their PCR efficiency in a four point standard curve and their specificity was evaluated on a gel as well as by performing a melting curve analysis. Primer sequences are provided in S3 Table. In order to determine relative gene expression, 2 μl of 1:1 diluted cDNA was used with 1 μM of each primer in SYBRgreen Real-Time PCR Master Mix (LifeTechnologies) on a 7900 HT Fast Real-Time PCR System machine (LifeTechnologies) with standard PCR conditions. The obtained C_t_ values were analyzed using the ΔΔCt method. Relative expression levels of the target genes *CDKN2A*, *BAX*, *CDKN1A*, *DRAM1*, *PHLDA1 SFN*, *YWHAB*, *YWHAE* and *YWHAZ* were normalized with up to three different housekeeping genes depending on the tissues analyzed—feathers: Eukaryotic translation elongation factor 2 (*EEF2*; [36]), Glyceraldehyde 3-phosphate dehydrogenase (*GAPDH*) and Ubiquitin (*UB*; [37]); skin: *EEF2*, *UB* and β*-actin* ([37]; liver: *EEF2* and β*-actin*; muscle: *EEF2* and β*-actin*). All samples were run in quadruplicates with target and housekeeping genes simultaneously. Unpaired Student’s t-test was used to assess the significance of the average expression values. As we were lacking a positive, apoptotic control sample for *BAX* qPCR experiments, we cannot formally exclude that the absence of *BAX* transcripts in our samples is due to technical errors. However, the primers were successfully used under similar conditions [38] and alternative methods did not suggest any ongoing apoptosis event in melanocytes.

### Genotyping and allelic imbalance

Four different pyro-sequencing assays were designed to properly determine the genotype at any of the four SNP positions associated with the Sex-linked barring phenotype. The primers were designed using PyroMark Assay Design 2.0 (Qiagen). cDNA or DNA samples were amplified as outlined in the Supplementary section. The obtained PCR products were purified and transferred to a PyroMark Q96 MD pyro-sequencing machine (Qiagen). In short, 20 μl of PCR product were hybridized to Streptavidin Sepharose High performance beads (GE Healthcare) and washed using 70% ethanol (Solveco), denatured in 0.2 M NaOH and washed again in wash buffer (composition as described in the pyro-sequencing manual) followed by a hybridization for 2 min incubation at 82°C.

### Immunohistochemistry and *in-situ* hybridization

For the immunohistochemistry analysis, primary antibodies were incubated overnight at 4°C, and secondary antibodies for 2 h at room temperature. Primary antibodies were against MITF (ab12039, Abcam), MART1 (ab731, Abcam) and Caspase-3 (Cleaved Caspase-3 (Asp175); Cell Signaling Technology). Alexa Fluor conjugated secondary antibodies were obtained from Invitrogen. Images were captured using a Zeiss Axioplan2 microscope equipped with Axiovision software (Carl Zeiss Vision GmbH). The statistical analysis was performed as One-way ANOVA; Tukey’s multiple comparison post-hoc test (n = 4).

Probes for *in-situ* hybridization detection of the *TYR* and *CDKN2A* transcripts (nucleotides 561–1168; NM_204160 and nucleotides 140–781; NM_204434 respectively) were generated by PCR using gene-specific primers (S3 Table) and cDNA obtained from chicken feathers in a reaction composed as followed: 1x KAPA2G GC buffer with 1.5 mM MgCl_2_ (KAPA Biosystems), 200 nM dNTPs, 200 pmol forward and reverse primer each with 1 U of KAPA2G Robust HotStart DNA Polymerase (KAPA Biosystems). The very same cycling program as described above was used with the only adjustment of 30 s for elongation time. PCR products were analyzed on an ethidium bromide stained low-melting-agarose gel and purified using the QIAquick Gel Extraction Kit (Qiagen). Quantity and quality of the obtained amplicons were measured using NanoDrop- 1000 Spectrophotometer (Thermo Scientific). Three μl of the purified PCR product were used for cloning into a pcDNA3.1/V5-His TOPO TA cloning vector (LifeTechnologies). The vector DNA from the clones was isolated using the QIAprep Miniprep Kit (Qiagen). To determine whether the insert was successfully cloned into the vector, the isolated plasmids were used directly in a PCR reaction with specific primers binding just outside the cloning site (T7 forward and BGH reverse provided with the cloning kit). As the procedure allows for insertion of the insert in both directions, the clones were sequenced and only antisense inserts were utilized to produce an *in-situ* probe.

*In-situ* hybridization analysis was performed as previously described [39]. In short, complementary RNA (cRNA) probes for ARF and TYR were made using the DIG RNA Labeling Kit (Roche Diagnostic GmbH). Probes were hybridized to untreated tissue sections overnight at 68°C under conditions containing 50% formamide and 5X Saline sodium citrate buffer in a humidified chamber. The DIG-labeled probes were detected using an alkaline phosphatase-conjugated anti-DIG antibody (Roche), followed by incubation with BCIP/NBT developing solution (Roche) for 2–5 h at 37°C. The intensity of the ISH-signal was analyzed using ImageJ (Rasband, W.S., ImageJ, NIH, Bethesda, Maryland, USA, http://imagej.nih.gov/ij/, 1997–2016). Bright field micrograph images were transformed to 8-bit grayscale and the overall intensity of the whole images was used for normalization and the mean intensity was analyzed after outlining each individual cell. Ten cells from each genotype were analyzed. One-way ANOVA with Tukey’s post hoc test, was used for statistical testing.

For both IHC and ISH four chickens/genotype were analyzed using four to six adjacent sections of two feathers shafts from the same individual.

### Circular dichroism spectra analysis and isothermal titration calorimetry

A truncated version of chicken MDM2 (NCBI accession number NP_001186313) containing amino acid residues 204–298 (MDM2_204-298_) was synthesized (GenScript) and cloned into the expression vector pSY5, a modified pET-21d(+) plasmid (Novagen), encoding an 8-histidine tag ahead of the N-terminus of the protein [40]. The protein was expressed in *Escherichia coli* strain BL21 (DE3) cells (Invitrogen) by inducing the cells at OD_600_ = 0.6 with 0.4 mM IPTG overnight at 16°C. All cells were grown in media containing 100 μg/ml ampicillin. Cells were harvested by centrifugation at 3,700 x g for 30 min. The pellet was subsequently resuspended in 100 ml binding buffer (50 mM Tris-HCl, pH 7.5, 500 mM NaCl, 20 mM imidazole), and disrupted by 2 mg/ml lysozyme treatment for 1 h at 4°C followed by sonication. Insoluble cell debris was removed by centrifugation at 32,000 x g for 45 min at 4°C. The supernatant was subjected to a multistep purification scheme using an ÄKTAxpress system (GE Healthcare), including (i) Ni^2+^ affinity chromatography (His-Trap FF 1 ml), in which the protein was eluted by an increasing concentration of imidazole; (ii) desalting (HiPrep 26/10) and ion exchange chromatography (Resource Q 1 ml) by using a buffer containing 50 mM Tris-HCl, pH 7.5 and eluting the protein with an increasing concentration of NaCl; and (iii) gel filtration chromatography (HiLoad 16/600 Superdex 200) equilibrated with 50 mM Tris-HCl, pH 7.5, 200 mM NaCl). The purified protein was concentrated to about 500 μM by using a centrifugal concentrator (Vivaspin 20, MWCO 3 kDa, Sartorius). Purity of the protein was checked by SDS-PAGE, identity by MALDI-TOF mass spectrometry (Bruker ultraflex TOF/TOF) and concentration estimated by a bicinchoninic acid assay (BCA; Thermo Scientific).

Peptides corresponding to the N-terminus of wild-type and mutant chicken ARF (residues 1–14; NCBI accession number AAN38848) were purchased from GL Biochem and denoted ARF_1-14_^WT^ ARF_1-14_^V9D^ and ARF_1-14_^R10C^, respectively. The concentrations of the peptides were estimated by measuring the free thiol groups in the peptides by mixing with 5,5-dithiobis-(2-nitrobenzoic acid) (DTNB, Ellman's reagent) in PBS and measuring the absorbance at 412 nm (extinction coefficient = 13.6 mM^-1^cm^-1^). All biophysical experiments were performed in PBS buffer, pH 7.3. Far-UV circular dichroism (CD) spectra between 200–260 nm were recorded on a Jasco J-810 spectropolarimeter (Jasco, Easton, MD) at 20°C using a cuvette with 1 mm path length. Four spectra were taken and averaged for 10 μM MDM2_204-298_ in presence or absence of 25 and 64 μM ARF_1-14_ peptide, respectively. The spectrum of buffer was subtracted from the protein/peptide spectra and the raw CD signal reported in mDeg.

Isothermal titration calorimetry (ITC) experiments were performed on a MicroCal iTC200 instrument (Malvern Instruments). The temperature during all experiments was 25°C. Before each ITC measurement, proteins were dialyzed using a dialysis cassette (Slide-A-Lyzer, MWCO 3.5 kDa, Thermo Scientific) against the experimental PBS buffer. The peptides were dissolved in the same dialyzing PBS buffer to reduce buffer mismatch in the ITC experiments. The background resulting from buffer to buffer titration was subtracted from the protein/peptide titration curve.

The ARF_1-14_^WT^ peptide, ARF_1-14_^V9D^ peptide or ARF_1-14_^R10C^ peptide (1.27 mM in the syringe) was titrated into MDM2_204-298_ (100 μM initial concentration in the cell), respectively. A titration usually consisted of one 0.5 μl injection followed by 19 injections of 2.0 μl. Because of the unknown but likely complicated mechanism of the ARF/MDM2 interaction [22], the experimental ITC data were not fit to a particular model but evaluated qualitatively.

### p53 reporter assay

U2 Osteosarcoma (U2OS) cells were obtained from Cell Line Service, Germany and cultured in Minimum Essential Media (LifeTechnologies) enriched with 10% Fetal Bovine Serum (LifeTechnologies) as well as 2 mM L-Glutamine (Sigma-Aldrich), 2,500 U of penicillin and 2.5 mg streptomycin (Sigma-Aldrich). The cells were kept at 37°C in a humidified atmosphere with 5% CO_2_. Media was changed every 2–3 days. Cells were sub-cultured at 90% confluence using 0.05% Trypsin-EDTA (LifeTechnologies). Cells were regularly tested for presence of Mycoplasma.

The ability of the missense mutations to affect p53 transcriptional activity through MDM2 interaction was tested in an assay as described [41]. A p53 reporter construct, p53-luc (Stratagene), containing several p53 responsive promoter elements fused to firefly luciferase, was used to assess p53 transcriptional activity following transfection with wild-type chicken ARF and mutant forms carrying the two different Sex-linked barring ARF missense mutations (V9D and R10C). The coding regions of the wild-type chicken ARF, V9D ARF and R10C ARF were cloned into pcDNA3.1 vector (Invitrogen, Life Technologies). U2OS cells were co-transfected at approximately 90% confluency with 1 μg of ARF plasmid, 1 μg p53-luc and 150 ng of phRG-Basic control *Renilla* plasmid (Promega) in 6-well plates utilizing 4 μl of Lipofectamine 2000 reagent (Invitrogen) in Opti-MEM medium (Gibco, Life Technologies, ThermoFisher Scientific). Five replicates for each construct were performed in three independent experiments. The cells were lysed 48 h post-transfection and firefly and *Renilla* luciferase activities were measured using the Dual Luciferase Reporter Assay System (Promega) with an Infinite M200 Luminometer (Tecan Munich GmbH). Firefly values were divided by *Renilla* values to normalize for fluctuations in plated cells and transfection efficiency. Relative luciferase units were then calculated by dividing the value of the different ARF forms by the value of the empty vector. Differences between the ARF versions were analyzed by Student’s t-test.

### Mycoplasma testing

In brief 1 ml of re-suspended cell suspension was used for DNA extraction using the culture cell protocol for the DNeasy Blood and Tissue Kit (Qiagen). The suspension was centrifuged for 3 min at 1,000 rpm to pellet the cells and re-suspended in 200 μl DPBS containing with 20 μl proteinase K. The protocol was then followed as described by Qiagen. The obtained DNA was tested for the presence of Mycoplasma DNA by using specific primers as well as the internal control in the Venor Germ Mycoplasma Detection Kit (Minerva Biolabs) with slight modifications. Only 0.5 U of Platinum Taq Polymerase (LifeTechnologies) were used per 25 μl reaction with 20–200 ng of DNA. The samples were amplified in an initial denaturing stage at 95°C for 5 min followed by 41 cycles at 95°C for 20 s, 55°C for 20 s, 72°C for 30 s and a final elongation time for 2 min at 72°C. Five μl PCR product were checked on an 3% agarose gel stained with ethidium bromide.

## Supporting information

S1 Table Average number of MITF+, MART1+, *TYR*+ and *CDKN2A*+ cells/mm^2^ in different parts of the feather in different genotypes. Significant differences from the value obtained for *N/N* are indicated by stars (One-way ANOVA, Tukey’s multi-comparison post-hoc test; * *P*<0.05, ** *P*<0.01, *** *P*<0.001).
(DOCX)

S2 Table *In situ* hybridization signal in chicken feather follicles obtained with a CDKN2A probe in different *CDKN2A* genotypes. (DOCX)

S3 Table Primer sequences used to investigate molecular mechanisms of *Sex-linked barring* mutations. (DOCX)

S1 Fig Phenotype of chicks at hatch with different *CDKN2A* genotypes: (A) *N/N*, (B) *B0/N* and (C) *B2/N* allele. The arrow marks the characteristic white spot associated with Sex-linked barring.
(TIF)

S2 Fig *B2/N* male feathers from different body regions. (TIF)

S3 Fig Relative expression of downstream targets of p53 (*CDKN1A*, *DRAM1* and *PHLDA3*) as well as four members of the 14-3-3 gene family involved in cell cycle regulation (*YWHAB*, *YWHAE*, *YWHAZ* and *SFN*). Significant differences in average relative gene expression between *B0/-* and *N/-* feathers were only observed for *PHLDA3* and *YWHAB*. Expression data was normalized using *EEF2* and *UB*. *P<0.05.
(TIF)

S4 Fig Caspase staining of different parts of the feather follicle for chickens carrying three different *CDKN2A* genotypes. No pre-apoptotic cells were observed in any feather region apart from the pulp.
(TIF)

S5 Fig Far-UV CD spectra of (A) MDM2_204-298_, ARF_1-14_^WT^ and ARF_1-14_^WT^/MDM2_204-298_ at different concentrations, (B) MDM2_204-298_, ARF_1-14_^V9D^/MDM2_204-298_ and ARF_1-14_^V9D^ at different concentrations and (C) MDM2_204-298_, ARF_1-14_^R10C^ and ARF_1-14_^R10C^/MDM2_204-298_ at different peptide concentrations.
(TIF)

S6 Fig Isothermal titration calorimetry experiments in which (A) ARF peptides WT, (B) V9D and (C) R10C were titrated into 100 μM MDM2_204-298_. Top panels, peaks resulting from heat of dilution upon titration of 1.27 mM into 100 μM. Middle panels, uncorrected peaks for titration of ARF peptides into MDM2_204-298_. Bottom panels, integrated heat data corrected for the heat of dilution.
(TIF)
