# Supplementary material for: The evolution of Sex-linked barring alleles in chickens involves both regulatory and coding changes in CDKN2A
Source: PLoS Genet. 2017 Apr 7;13(4):e1006665. doi: 10.1371/journal.pgen.1006665 (PMC5384658; doi:10.1371/journal.pgen.1006665)
Supplement: S1 Table — Significant differences from the value obtained for N/N are indicated by stars (One-way ANOVA, Tukey’s multi-comparison post-hoc test; * P<0.05, ** P<0.01, *** P<0.001). (DOCX) [file pgen.1006665.s001.docx]

**S1 Table.** Average number of MITF+, MART1+, *TYR*+ and *CDKN2A*+ cells/mm^2^ in different parts of the feather in different genotypes. Significant differences from the value obtained for *N/N* are indicated by stars (one-way ANOVA, Tukey’s multi-comparison post-hoc test; * *P*<0.05, ** *P*<0.01, *** *P*<0.001).

| Region^a^ | MITF cells/mm^2^ | | |  | Region^a^ | MART1 cells/ mm^2^ | | | | | | |
| --- | --- | --- | --- | --- | --- | --- | --- | --- | --- | --- | --- | --- |
|  | ***N/N*** | ***B0/W*** | ***B2/N*** |  |  | ***N/N*** | ***B0/W*** | | | ***B2/N*** | | |
| BA | 3470±454 | 1070±419** | 2090±648* |  | **BA** | 1970±207 | | | 790±209*** | | | 1740±109 |
| RGZ | 2500±246 | 742±442*** | 1650±618 |  | **RGZ** | 1180±159 | | | 642±207 | | | 798±326 |
| UB | 1160±132 | 0±0* | 675±273 |  | **UB** | 0±0 | | | 276±76 | | | 484±203 |
| MB | 625±84 | 0±0 | 0±0 |  | **MB** | 0±0 | | | 0±0 | | | 0±0 |
| LB | 69±120 | 0±0 | 0±0 |  | **LB** | 0±0 | | | 0±0 | | | 0±0 |
| PE | 0±0 | 0±0 | 0±0 |  | **PE** | 0±0 | | | 0±0 | | | 0±0 |
|  |  |  |  |  |  |  | | |  | | |  |
|  | ***TYR* cells/mm^2^** | | |  |  | ***CDKN2A* cells/mm^2^** | | | | | | |
|  | ***N/N*** | ***B0/W*** | ***B2/N*** |  |  | ***N/N*** | | ***B0/W*** | | | ***B2/N*** | |
| BA | 2130±323 | 481±113*** | 1310±531 |  | **BA** | 292±70 | | 1130±345* | | | 920±194* | |
| RGZ | 1270±238 | 398±176** | 1060±256 |  | **RGZ** | 0±0 | | 246±213 | | | 155±136 | |
| UB | 0±0 | 104±181 | 0±0 |  | **UB** | 0±0 | | 0±0 | | | 0±0 | |
| MB | 0±0 | 0±0 | 0±0 |  | **MB** | 0±0 | | 0±0 | | | 0±0 | |
| LB | 0±0 | 0±0 | 0±0 |  | **LB** | 0±0 | | 0±0 | | | 0±0 | |
| PE | 0±0 | 0±0 | 0±0 |  | **PE** | 0±0 | | 0±0 | | | 0±0 | |
| ^a^BA=barb, RGZ=ramogenic zone, UB=upper bulge, MB=middle bulge, LB=lower bulge, PE=papilla ectoderm. | | | | | | | | | | | | |
