# Supplementary material for: The evolution of Sex-linked barring alleles in chickens involves both regulatory and coding changes in CDKN2A
Source: PLoS Genet. 2017 Apr 7;13(4):e1006665. doi: 10.1371/journal.pgen.1006665 (PMC5384658; doi:10.1371/journal.pgen.1006665)
Supplement: S2 Table — (DOCX) [file pgen.1006665.s002.docx]

**S2 Table.** *In-situ*-hybridization signal in chicken feather follicles obtained with a *CDKN2A* probe in different *CDKN2A* genotypes. There were no statistically significant differences between genotypes (one-way ANOVA multi-comparison Tukey’s posthoc test).

| Genotype | n | Intensity^a^ |
| --- | --- | --- |
| *N/N* | 10 | 127±13.0 |
| *B0/W* | 10 | 126±6.6 |
| *B2/N* | 10 | 131±6.6 |

n = number of cells analyzed

^a^Mean±SE
