# Supplementary material for: The evolution of Sex-linked barring alleles in chickens involves both regulatory and coding changes in CDKN2A
Source: PLoS Genet. 2017 Apr 7;13(4):e1006665. doi: 10.1371/journal.pgen.1006665 (PMC5384658; doi:10.1371/journal.pgen.1006665)
Supplement: S3 Table — (DOCX) [file pgen.1006665.s003.docx]

**S3 Table.** Primer sequences used to investigate molecular mechanisms of *Sex-linked barring* mutations.

| Application | Gene | Forward primer  5->3’ | Reverse primer  5->3’ | Sequencing primer  5->3’ |
| --- | --- | --- | --- | --- |
| qPCR | ***CDKN2A*** | AGGGTGGAGAACATCAAACA | AGCGGCTGTCTCACTTCT | None |
|  | ***CDKN1A*** | GGGCAGACCACCATCAA | ATCCTCAGCAGCAGCAGTC | None |
|  | ***BAX^D^*** | acagggtttcatccaggatcgagca | tcagcttcttggtggacgcatc | None |
|  | ***DRAM1*** | CATGACAAAAATTGATTGGACTC | CCACCGTCCATTCACAGAT | None |
|  | ***PHLDA3*** | GTGGTGATGGACGACAGC | CATGGTGATGGCAGCATT | None |
|  | ***SFN*** | CCAGCATCGAGCACAAAA | TTCAGCTCCTCTTCCACCTT | None |
|  | ***YWHAB*** | TCAATGGAGTTGCAACAGGT | TACTCCACCGAGCCTTCTTT | None |
|  | ***YWHAE*** | CCTCATTCCAGCAGCTAACA | TGGCAAACTCAGCCAGATAC | None |
|  | ***YWHAZ*** | CCTTCTCTGGGGAATTGAGA | AAAAAGGAGATGCAGCCAAC | None |
|  | ***EEF2***^A^ | GCACGTGGATTTCTCTTCAG | CAGGTTTGATCCTCTCAGCA | None |
|  | ***GAPDH*** | GGTGAAAGTCGGAGTCAACGG | TCGATGAAGGGATCATTGATGGC | None |
|  | ***UB***^B^ | GGGATGCAGATCTTCGTGAAA | CTTGCCAGCAAAGATCAACCTT | None |
|  | ***Actin***^C^ | CACAGATCATGTTTGAGACCTT | CATCACAATACCAGTGGTACG | None |
| Pyro | **SNP1** | AGCGACAGTCCGCAACATAAAATAA | CACGACGTTGTAAAACGACTTCCCGATCTCGCAGGCTT | AAAAAGATCGTCCTTCTG |
|  | **SNP2** | TTCTGTTCTAAATATAAATGCAACTCG | CACGACGTTGTAAAACGACTTTGCACTTCGTTTTCTTTTTGA | ACCGTCTGGGCGTTC |
|  | **SNP3/**  **SNP4** | CACGACGTTGTAAAACGACTCTCCGGGACCTCTCCTGT | GAGCAGCGAGAAGGACAGAG | TGCGGGCGCGCCGAA |
| In-situ | ***CDKN2A*** | TGTTCCCATGACCTCTCGGATAAGG | GCAACCGACGGAATGTTTGATGTT | None |
|  | ***TYR*** | TGCTACTGGCACATATGCTCAGATGAAC | TTGCAGTGTGTGGATCAGCAAAGC | None |

^A^ Kouadjo et al. [41]; ^B+C^De Boever et al. [42]; ^D^ Li et al. [43]
