# Supplementary figures and images for: The evolution of Sex-linked barring alleles in chickens involves both regulatory and coding changes in CDKN2A
Source: PLoS Genet. 2017 Apr 7;13(4):e1006665. doi: 10.1371/journal.pgen.1006665 (PMC5384658; doi:10.1371/journal.pgen.1006665)

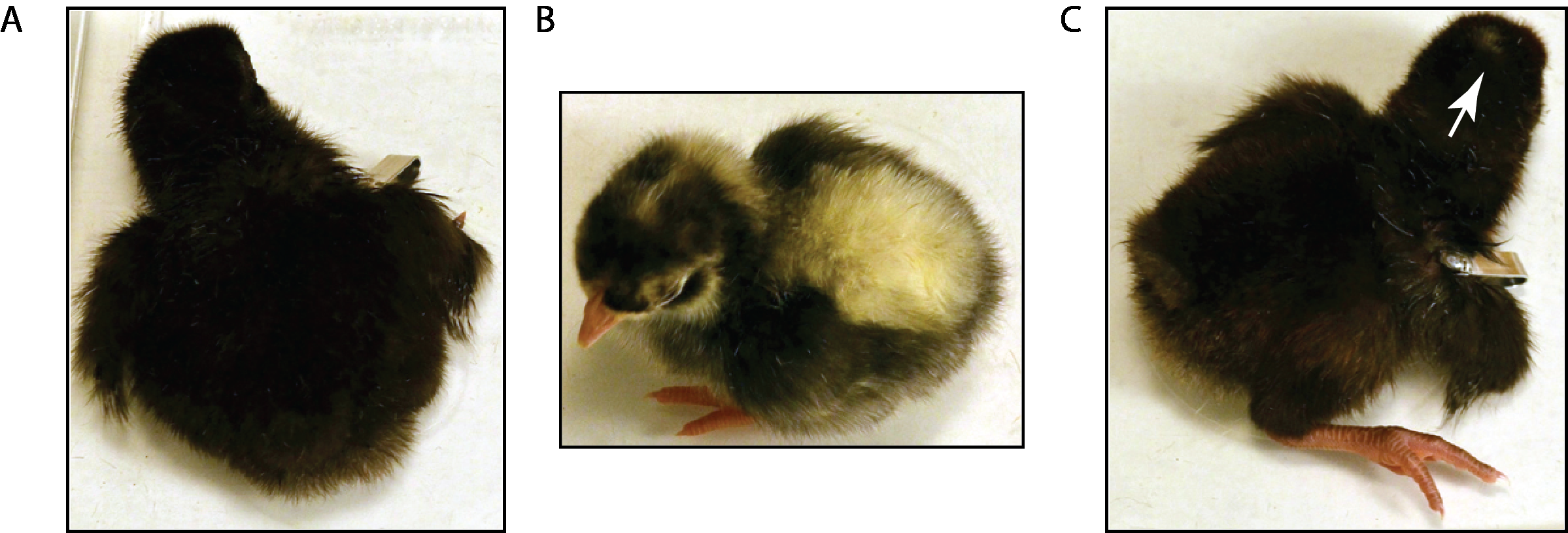

Supplement: S1 Fig — Phenotype of chicks at hatch with different CDKN2A genotypes: (A) N/N, (B) B0/N and (C) B2/N allele. The arrow marks the characteristic white spot associated with Sex-linked barring. (TIF) [file pgen.1006665.s004.tif]

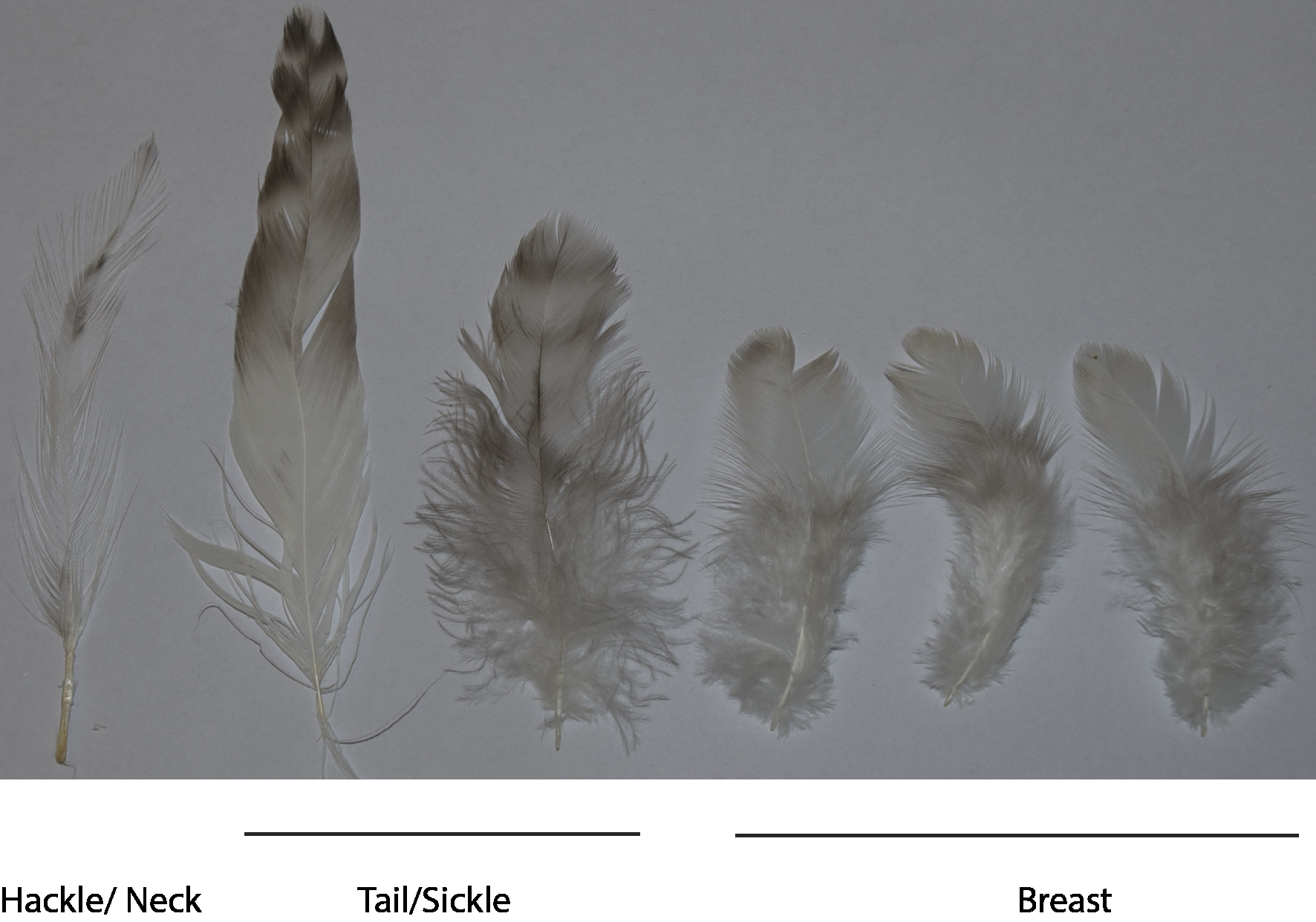

Supplement: S2 Fig — (TIF) [file pgen.1006665.s005.tif]

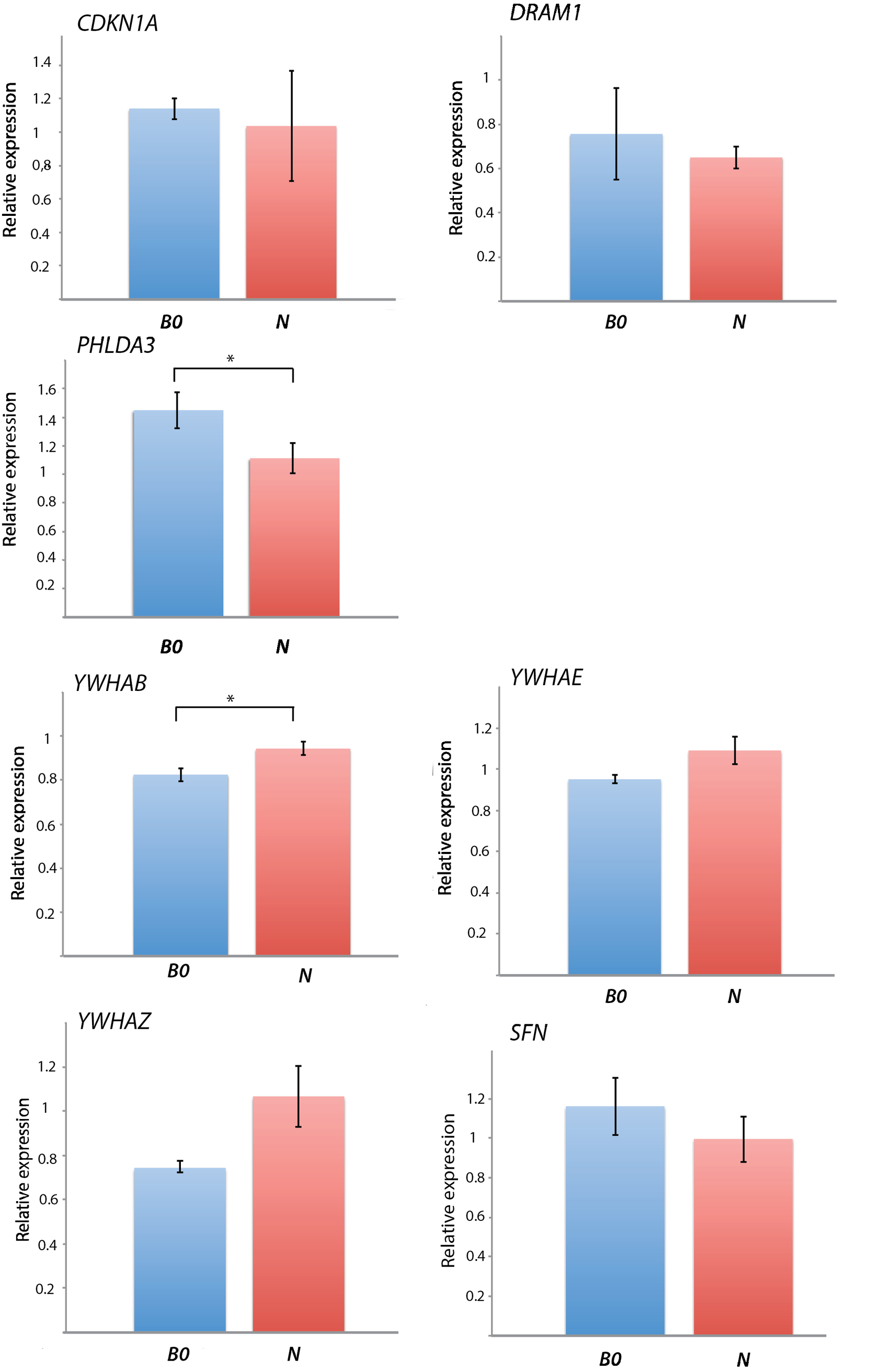

Supplement: S3 Fig — Significant differences in average relative gene expression between B0/- and N/- feathers were only observed for PHLDA3 and YWHAB. Expression data was normalized using EEF2 and UB. *P<0.05. (TIF) [file pgen.1006665.s006.tif]

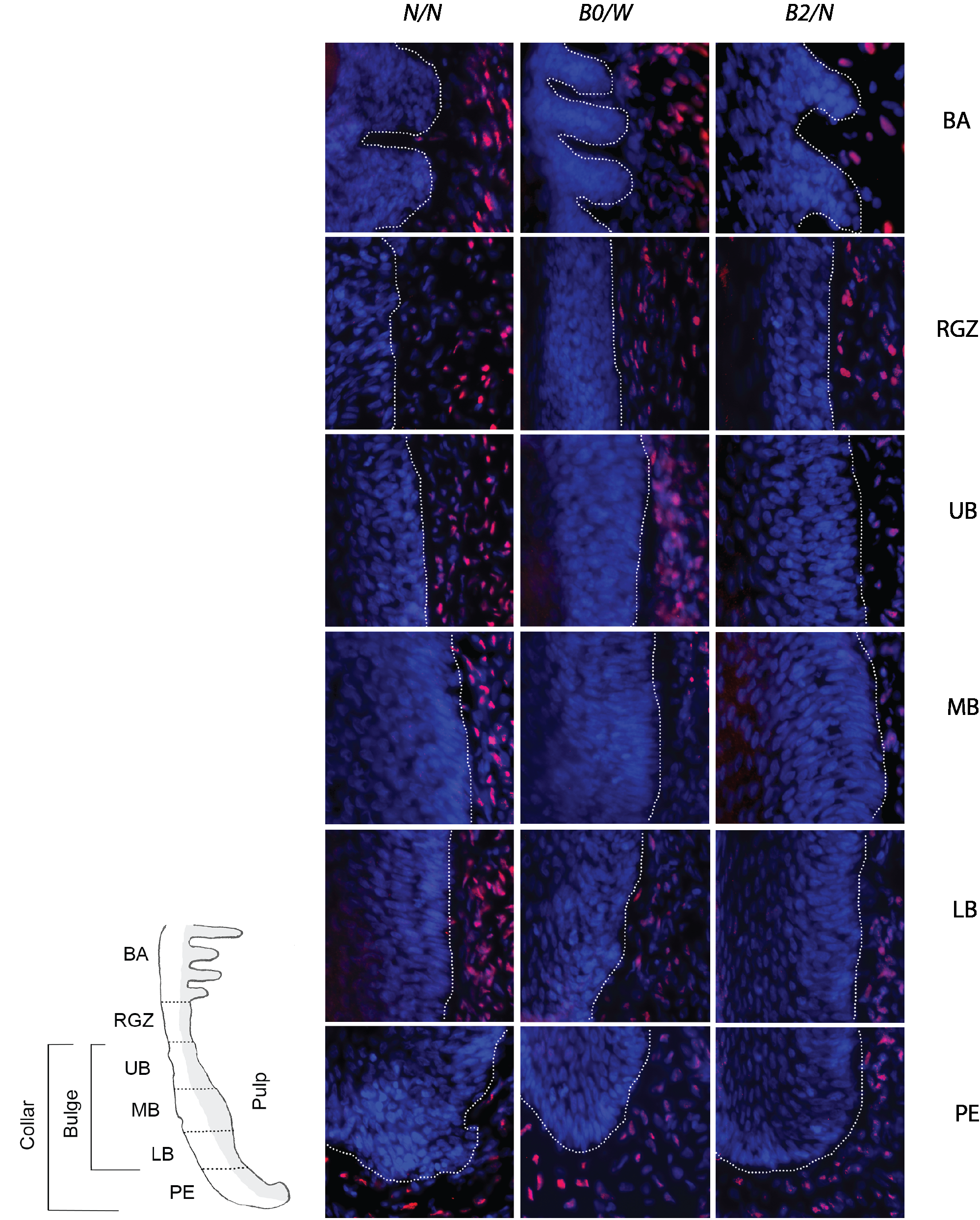

Supplement: S4 Fig — No pre-apoptotic cells were observed in any feather region apart from the pulp. (TIF) [file pgen.1006665.s007.tif]

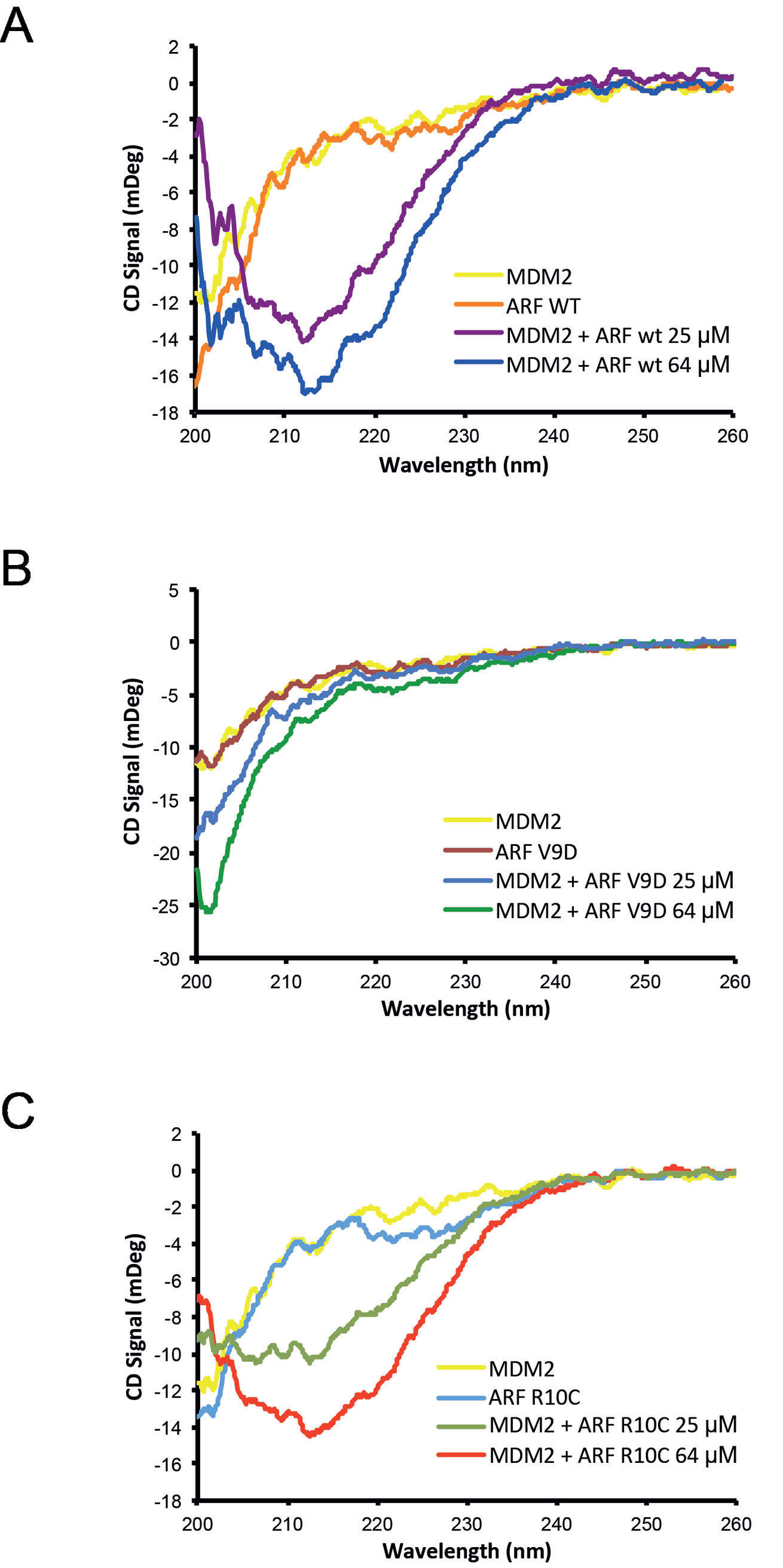

Supplement: S5 Fig — Far-UV CD spectra of (A) MDM2204-298, ARF1-14WT and ARF1-14WT/MDM2204-298 at different concentrations, (B) MDM2204-298, ARF1-14V9D/MDM2204-298 and ARF1-14V9D at different concentrations and (C) MDM2204-298, ARF1-14R10C and ARF1-14R10C/MDM2204-298 at different peptide concentrations. (TIF) [file pgen.1006665.s008.tif]

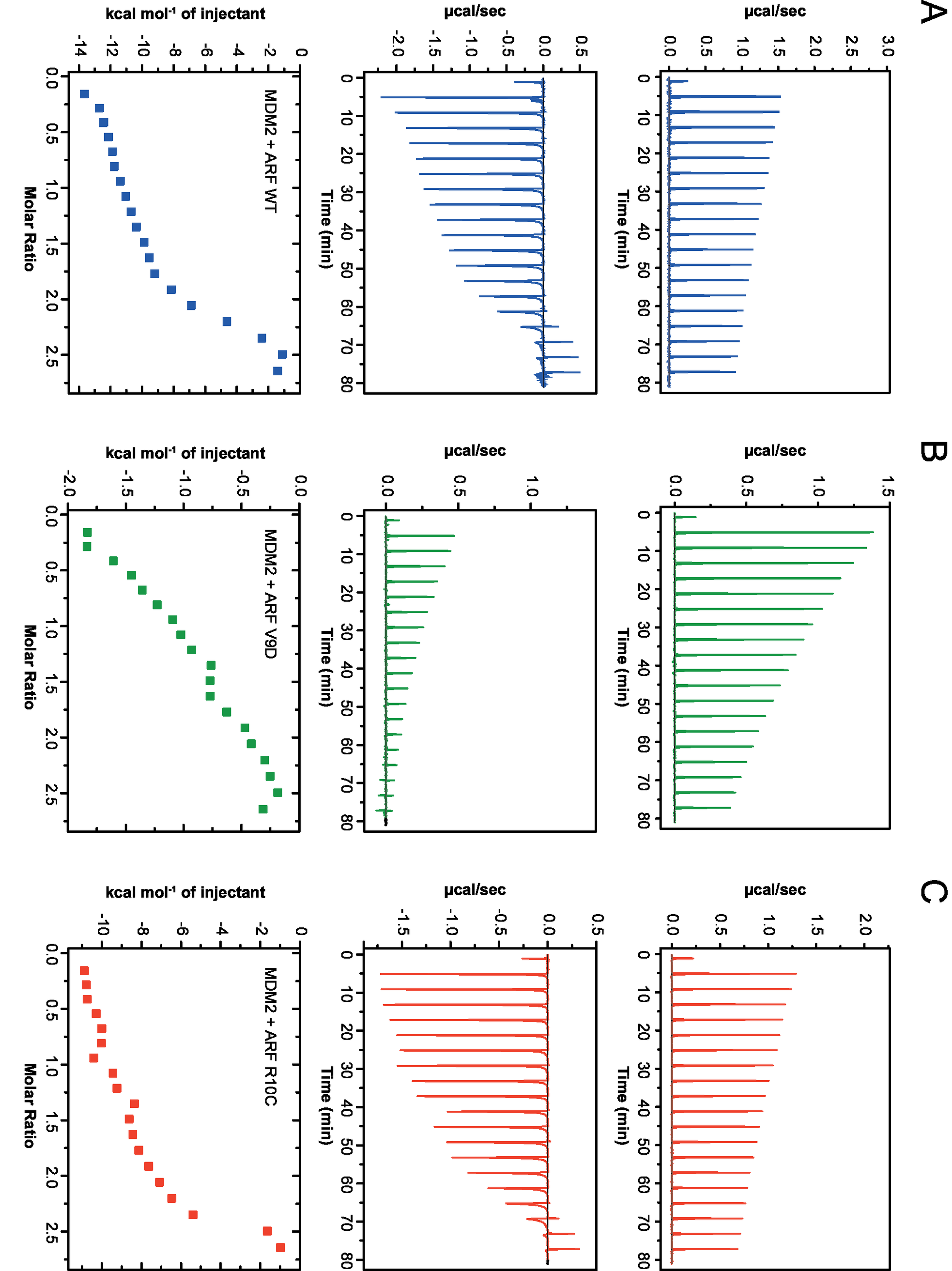

Supplement: S6 Fig — Isothermal titration calorimetry experiments in which (A) ARF peptides WT, (B) V9D and (C) R10C were titrated into 100 μM MDM2204-298. Top panels, peaks resulting from heat of dilution upon titration of 1.27 mM into 100 μM. Middle panels, uncorrected peaks for titration of ARF peptides into MDM2204-298. Bottom panels, integrated heat data corrected for the heat of dilution. (TIF) [file pgen.1006665.s009.tif]
